# Circadian and ultradian rhythms of clock gene expression in the suprachiasmatic nucleus of freely moving mice

**DOI:** 10.1038/srep12310

**Published:** 2015-07-21

**Authors:** Daisuke Ono, Ken-ichi Honma, Sato Honma

**Affiliations:** 1 Photonic Bioimaging Section, Research Center for Cooperative Projects, Hokkaido University Graduate School of Medicine, Sapporo, 060-8638, Japan; 2 Department of Chronomedicine, Hokkaido University Graduate School of Medicine, Sapporo, 060-8638, Japan

## Abstract

In mammals, the temporal order of physiology and behavior is primarily regulated by the circadian pacemaker located in the hypothalamic suprachiasmatic nucleus (SCN). Rhythms are generated in cells by an auto-regulatory transcription/translation feedback loop, composed of several clock genes and their protein products. Taking advantage of bioluminescence reporters, we have succeeded in continuously monitoring the expression of clock gene reporters *Per1-luc*, PER2::LUC and *Bmal1-ELuc* in the SCN of freely moving mice for up to 3 weeks in constant darkness. Bioluminescence emitted from the SCN was collected with an implanted plastic optical fiber which was connected to a cooled photomultiplier tube. We found robust circadian rhythms in the clock gene expression, the phase-relation of which were the same as those observed *ex vivo*. The circadian rhythms were superimposed by episodic bursts which had ultradian periods of approximately 3.0 h. Episodic bursts often accompanied activity bouts, but stoichiometric as well as temporal analyses revealed no causality between them. Clock gene expression in the SCN *in vivo* is regulated by the circadian pacemaker and ultradian rhythms of unknown origin.

The circadian clock in mammals is a hierarchical multi-oscillator system. The master pacemaker is located in the hypothalamic suprachiasmatic nucleus (SCN) and its outputs entrain peripheral oscillators in the extra-SCN tissues^1^. The SCN circadian pacemaker is a self-sustained oscillator where clock genes *Per1*, *Per2*, *Cry1*, *Cry2, Clock* and *Bmal1* play crucial roles. Heterodimers of *Clock* and *Bmal1* proteins (CLOCK/BMAL1) activate the transcription of the *Per* and *Cry* genes, the protein products of which in turn suppressed the transactivation of CLOCK/BMAL1, closing this feedback loop^2^. One turn of this auto-feedback loop takes approximately 24 hr. The core clock gene mechanism is sensitive to external cues, such as light that can reset the clock^3^ as well as internal cues such as behavior that can modulate clock function^4^. Thus, a full and accurate picture of circadian clock dynamics would be understood in the intact system.

Substantial knowledge on the pacemaker dynamics in the SCN has been obtained by real time monitoring of clock gene expression in cultured slices or dispersed cells^5^,^6^. However, the effects of slicing and culturing on circadian oscillation in the SCN are not well understood. Moreover, effects of inputs from other brain areas on the SCN and how the clock genes respond to them are totally unknown. *In vivo* monitoring of clock gene expression in the SCN of freely moving animals may solve these questions and reveal relationships between clock gene expressions in the SCN and its outputs. Despite one pioneering study in which circadian rhythms in clock gene expression were monitored *in vivo* using a bioluminescence gene reporter^7^, we are still ignorant of the clock gene dynamics in the intact SCN of freely moving animals. By employing a highly sensitive bioluminescence recorder, we have overcome technical barriers that have slowed progress in the SCN *in vivo*.

## Results

### Measurement of bioluminescence from the SCN of freely moving mice

**Figure 1:**
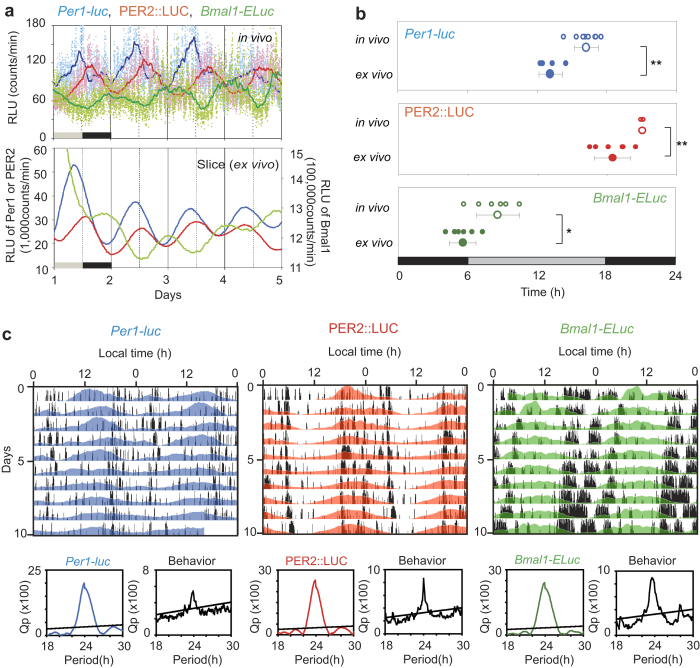
Circadian rhythms in clock gene bioluminescence from the SCN and in behavior in freely moving mice (**a**) Typical examples of *Per1-luc* (blue), PER2::LUC (red), and *Bmal1-ELuc* (green) rhythms in the SCN *in vivo* (upper) and *ex vivo* (lower). *In vivo* bioluminescence is plotted as raw counts at one min intervals (dots) and as a 4 h moving average (solid lines). *Ex vivo* bioluminescence is plotted at 10 min intervals as a 50 min moving average. Vertical lines in each panel indicate local times (solid line, 06:00 h; broken line, 18:00 h). (**b**) Circadian peak phases of *Per1-luc* (blue), PER2::LUC (red), and *Bmal1-ELuc* (green) *in vivo* (open circle) and *ex vivo* (closed circle) in the first circadian cycle (Day 1 in constant darkness (DD)) for the *in vivo* experiment and on Day 1 of culturing for the *ex vivo* experiment. Smaller circles indicate individual peaks and larger circles with a bar indicate the group means and SD. Grey and black bars at the bottom of each panel indicate the antecedent light-dark (LD) cycle (black: dark phase) before transfer to DD. Student’s t-test: *, <0.05; **, <0.01. (**c**) Representative examples of *in vivo* circadian *Per1-luc* (blue), PER2::LUC (red) or *Bmal1-ELuc* (green) rhythms in the SCN are illustrated with behavior (black) in actograms. Chi square periodograms for clock gene reporter and behavior activity are demonstrated under each actogram with the same colors as in the actogram. An oblique line in the periodogram indicates a significance level of p = 0.05. Each line shows the gene expression and activity distribution across a day, and sequential days are plotted from top to bottom. For better visualization, actograms are double plotted (48-h x axis). *In vivo* circadian rhythms are smoothed by a 4 h moving average method and detrended by a 24 h moving average subtraction method.

In the present study, several technical improvements were made to enable bioluminescence recordings of the SCN in freely moving mice (Fig. 1, Supplementary Figure S1). First, cooling the photomultiplier tube (PMT) in the photon counting devise (*In vivo* Kronos, Atto) to 10 °C reduced dark counts and consequently increased the signal to noise ratio. Background noise was reduced from 834.2 ± 91.0 to 36.2 ± 6.5 counts/min (mean ± SD) and became stable (Supplementary Figure S1). In addition, a convex lens (Plano-Convex lens, #45-081, Edmond) between the optical fiber and PMT optimized the detection area and increased signal strength by ca 40%. Second, the substrate luciferin was delivered to the SCN via an osmotic pump implanted in the body, instead of perfusing it through the lateral ventricle^7^. Third, to ensure that the mice could move freely, a long (3 m) plastic optical fiber was employed in order to reduce fiber torque. These improvements all together enabled us to monitor bioluminescence in the SCN of freely moving mice up to 3 weeks (Supplementary Figure S1e).

### Circadian rhythms in *Per1-luc*, *Bmal1-ELuc* and PER2::LUC in the SCN of freely moving mice

**Figure 2:**
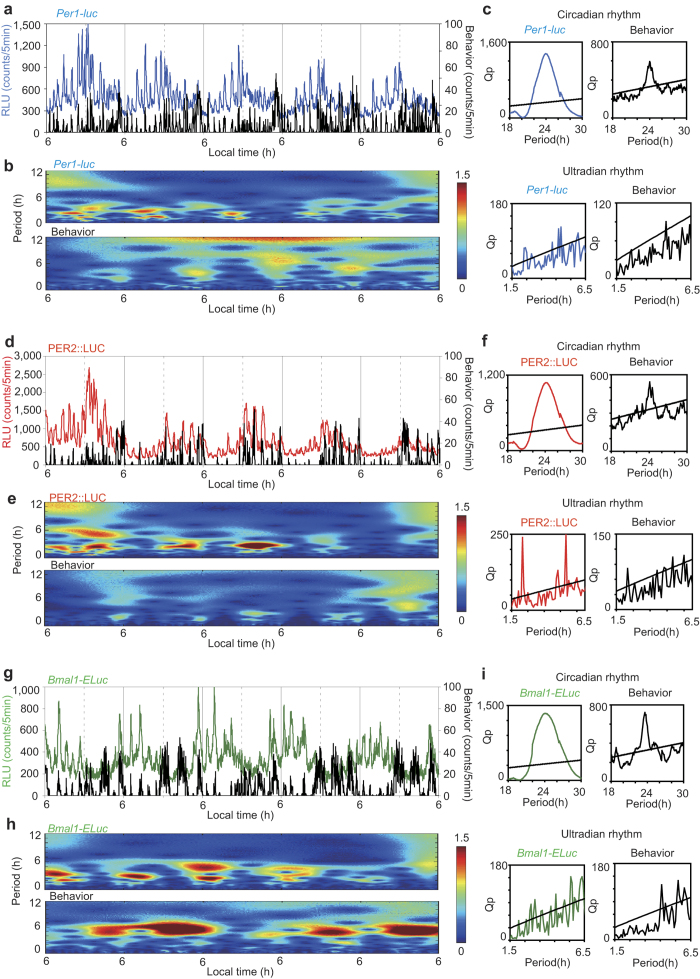
Episodic bursts of reporter bioluminescence and activity bouts *in vivo* (**a**, **d**, **g**) Representative episodic fluctuations of reporter bioluminescence (colored traces) and simultaneously measured activity bouts (black traces) in freely moving mice for 5 consecutive days (a, *Per1-luc*; d, PER2::LUC; g, *Bmal1-ELuc*). Raw data (non-deterended, non-smoothed) are plotted in 5 min bins. (**b**, **e** ,**h**) Wavelet spectrum is illustrated for episodic bursts of bioluminescence (upper) and for activity bout (lower) in the ultradian range (0-12 h). The intensity of rhythm at a particular period was expressed with different colors. Dark red indicates the intensity larger than 1.5 time of the individual mean peak level of bioluminescence burst (Fig. 4). Intensities below this level are expressed by different colors down to dark blue. (**c**, **f**, **i**) Chi square periodograms in the circadian (upper) and ultradian ranges (lower) are illustrated for bioluminescence (left) and behavior activity (right) in each clock gene with the same colors as in the actogram. An oblique line in each periodogram represents a significance level (p < 0.05).

Circadian rhythms in clock gene expression were observed continuously for up to 3 weeks in the SCN of freely moving mice, carrying bioluminescence reporters for the expression of *Per1* (*Per1-luc*), *Bmal1* (*Bmal1-ELuc*) or for the protein product of *Per2* (PER2::LUC) (Fig. 1, Fig. 2, Supplementary Figure S1, Figure S2). Histological examination revealed that the tip of an implanted optical fiber was located at the dorsal border of the SCN in most cases and was found within the dorsal part of the SCN in the rest of cases (Supplementary Figure S1c).

The bioluminescence detected by the fiber originated mainly from the dorsal area of the SCN, because only 30% (*Bmal1-ELuc*) to 50% (*Per1-luc*, PER2::LUC) of bioluminescence emitted from the ventral area of the SCN was estimated to reach the optical fiber^8^. The difference between the clock gene reporters is due to the wavelength of lights emitted by the reporters (561 nm vs. 630 nm). The spatial distribution profiles of bioluminescence intensity were obtained using data from cultured coronal slices of the SCN prepared from the same clock gene reporter mice (Supplementary Figure S3). The intensity of bioluminescence was brightest at the medial part of the SCN, whereas the intensity at the border of SCN was less than 10% of the brightest area in the SCN and decreased rapidly with distance from the SCN border.

Significant circadian rhythms were detected in clock gene expressions by Chi square periodogram (p < 0.05) in all mice whose SCN emitted measureable bioluminescence (Fig. 1a). Simultaneously measured behavior activity by a passive infrared sensor also showed significant circadian rhythms (Fig. 1c). Estimates of the circadian period in gene expression rhythm were slightly different among the three strains of mice but not between gene expression rhythms and their respective behavior rhythms (*Per1-luc* mice, 23.7 ± 0.3 h for bioluminescence and 23.9 ± 0.2 h for behavior activity, n = 8; PER2::LUC mice, 24.3 ± 0.3 h and 24.0 ± 0.3 h, n = 3; *Bmal1-ELuc* mice, 23.9 ± 0.1 h and 23.9 ± 0.2 h, n = 7, by Chi square periodogram).

The circadian rhythms in clock gene expression measured *ex vivo* were significantly phase-advanced by approximately 3 hours in all 3 clock gene reporters. Nonetheless, the phase-relationship among the *Per1*, PER2 and *Bmal1* is consistent with results from *in situ* hybridization^9^,^10^ and in *ex vivo* studies^11^,^12^,^13^.

### Episodic bursts of clock gene reporters in the SCN and ultradian rhythmicity

Unexpectedly, the *in vivo* circadian rhythms in gene expression were superimposed by episodic bursts of bioluminescence in all three reporters (Fig. 2, Supplementary Figure S2). Chi square periodogram revealed significant ultradian periods in all mice. Most mice showed more than one periodicity of different strength in the ultradian range. The major period was 3.0 ± 0.5 h (n = 8) for *Per1-luc*, 2.9 ± 0.3 h (n = 3) for PER2::LUC, and 3.2 ± 0.8 h (n = 7) for *Bmal1-ELuc*, respectively. Wavelet analysis confirmed these ultradian periodicities (Fig. 2, Supplementary Figure S2). Ultradian rhythms were also detected in behavior activity by Chi square periodogram in all mice examined (4.7 ± 0.2 h, n = 8, *Per1-luc*; 3.7 ± 0.9 h, n = 3, PER2::LUC; 4.6 ± 1.1 h, n = 7, *Bmal1-ELuc*), and the periods were slightly but significantly longer than those observed in clock genes (p < 0.01, *Per1-luc*; p < 0.05, PER2::LUC; p < 0.05, *Bmal1-ELuc*, one-way repeated measure ANOVA).

**Figure 3:**
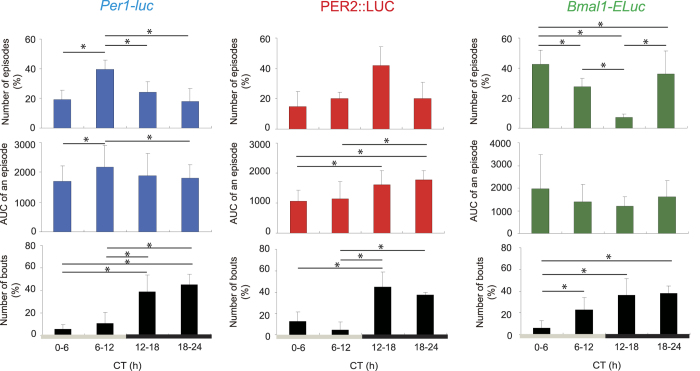
Dependency of episodic bursts and activity bouts on circadian rhythm Dependence of the number (upper panels), and size (AUC) of bioluminescence bursts (middle panels) as well as the number of activity bouts (lower panels) on the circadian phase of the respective clock gene. The circadian phase was divided into 4 phases based on CT (CT0-6, early subjective day; CT6-12, late subjective day; CT12-18, early subjective night; CT18-24, late subjective night). Blue, red and green columns with a vertical bar (mean ± SD) indicate the results of *Per1-luc*, PER2::LUC and *Bmal1-ELuc* reporter, respectively. Gray and black bars at the bottom of the panels indicate the subjective day and night. Asterisk (*) indicates statistically significant difference between the values in different phases (p < 0.05, one-way repeated measure ANOVA with a post-hoc Tukey-Kramer).

**Figure 4:**
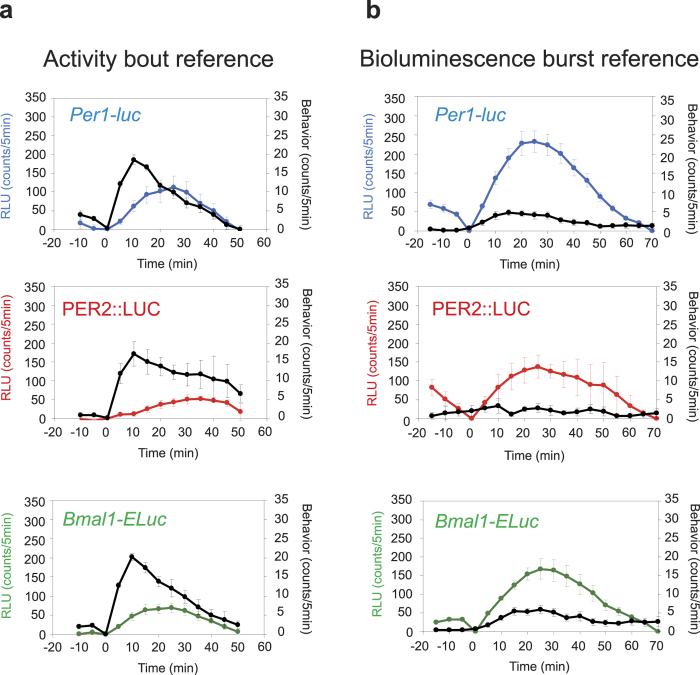
Stoichiometric relations between episodic burst of bioluminescence and activity bout (**a**) Temporal relations between the mean activity bout and accompanying bioluminescence (behavior bout reference) are illustrated for each clock gene reporter. (**b**) Temporal relations between the mean episodic burst of bioluminescence and accompanying behavior activity (bioluminescence episodic reference) are illustrated for each clock gene reporter. The mean values were obtained by adjusting individual times of initial trough or of 0 counts. Values were expressed as mean ± SEM.

The frequency and size of episodic bursts were examined to characterize episodic bursts of clock gene reporters. Episodic bursts occurred either sporadically or sequentially. In the latter case, bursts overlapped, leading a stepwise increase in bioluminescence that lasted up to several hours. Since most sporadic episodes persisted for approximately one hour, episodic bursts of less than 2 h duration were used for further analyses. A total of 691 episodic bursts were detected during the first 5 circadian cycles under DD for all mice (*Per1-luc*, n = 342; PER2::LUC, n = 106; *Bmal1-ELuc*, n = 243). The mean number of episodic bursts per mouse was 42.8 ± 10.8/5 cycles for *Per1-luc*, 35.3 ± 20.7/5 cycles for PER2::LUC and 34.7 ± 8.0/5 cycles for *Bmal1-ELuc*. The mean duration of episodic burst was 70.5 ± 7.5 min for *Per1-luc*, 85.0 ± 5.0 min for PER2::LUC and 75.5 ± 9.5 min for *Bmal1-ELuc*, respectively. The frequency and the size of episodic burst depended on the circadian phase of respective reporter genes (Fig. 3). The size of episode was expressed as an area under the curve (AUC). AUC was calculated by summing bioluminescence above the baseline. Both the number of episode and AUC of *Per1-luc* were largest in the late subjective day (CT6-CT12), those of PER2::LUC were in the early subjective night (CT12-CT18), and of *Bmal1-ELuc* in the early subjective day (CT0-CT6), respectively (p < 0.05, one-way repeated measure ANOVA), where CT12 was defined as the onset of behavior activity in terms of circadian time (CT) which was a time unit divided by a circadian period. The mean interval from the initial trough of a burst to the episodic peak was 25 min, irrespective of reporter gene (Fig. 4).

An episodic burst of bioluminescence was often accompanied by a bout of behavior activity (activity bout). To know whether there is causality between episodic bursts of bioluminescence and activity bouts or not, we examined their temporal as well as stoichiometric relationships. Activity bouts were detected mainly in the subjective night (CT12-CT24) (one-way repeated measure ANOVA, p < 0.01), irrespective of reporter gene (Fig. 3). Therefore, the peak times of bioluminescence bursts and activity bouts were antiphasic for *Bmal1-ELuc* and substantially out of phase for *Per1-luc*. The desynchronized phase relationship between episodic bursts and activity bouts was also revealed by wavelet analysis (Fig. 2, Supplementary Figure S2). The intensity of ultradian rhythms fluctuated in a circadian fashion in both measures, but the peak intensity was out of phase in *Per1-luc* and *Bmal1-ELuc*. Thus, there was no temporal correlation in the frequency or in the AUC between episodic bursts of bioluminescence and activity bouts at least in *Per1-luc* and *Bmal1-ELuc*.

The mean sizes of activity bout and accompanying bioluminescence or of episodic burst and accompanying behavior activity were compared to examine stoichiometric relations between the two measures (Fig. 4). In the activity bout reference, the mean activity bout peaked after 10 min at 17.1 to 20.2 counts/5 min. Accompanying bioluminescence started to increase almost simultaneously with an activity bout and peaked in 25 min (*Per1*, *Bmal1*) or in 35 min (PER2) on average. However, the peak bioluminescence level was variable (52.3–112.0 counts/5 min). By contrast, in the bioluminescence burst reference, the mean episodic burst peaked in 25 min in all gene reporters and the mean peak level was 138–233 counts/5 min, significantly larger than those obtained with the activity bout reference by 2–3 times, irrespective of the clock genes (p < 0.01, one-way repeated measure ANOVA). On the other hand, the mean peak levels of accompanying behavior activity (3–6 counts/5 min) was approximately one thirds of the peak levels obtained with the activity bout reference, and significantly lower than those, irrespective of the clock genes (p < 0.01, one-way repeated measure ANOVA). The behavior activity did not increase significantly in parallel with the episodic burst of PER2::LUC. Correlation analysis failed to reveal any significant relationship with respect to AUC and the peak level between activity bouts and accompanying bioluminescence or between episodic bursts of bioluminescence and accompanying behavior activity in all clock gene reporters. Thus, there was no evidence for a stoichiometric correlation between the episodic burst and the activity bout.

## Discussion

Circadian rhythms in *Per1-luc*, PER2::LUC and *Bmal1-ELuc* in the SCN of freely moving mice were measured continuously for up to 3 weeks using an *in vivo* monitoring system. The circadian period of each reporter matched the period of behavior *in vivo*, indicating that the reporter rhythms reflected adequately the circadian oscillation. Bioluminescence recorded by the present *in vivo* measurement likely drives primarily from the dorsal area of the SCN, since the intensity of bioluminescence at the ventral area of the SCN is estimated to decay by 70% (*Bmal1-ELuc*) or 50% (*Per1-luc*, PER2::LUC)^8^. Contamination of the signal by peri-SCN bioluminescence seems to be negligible, since the bioluminescence intensity at the border of SCN or outside the nucleus was less than 10% of the brightest area in the SCN (Supplementary Figure S3). The value was not large enough to modify episodic bursts, taking a 2 to 3 fold fluctuation range of bioluminescence from the circadian baseline into account. The circadian peaks of clock gene bioluminescence were located at different times of day for the three reporters, and almost antiphasic between PER2::LUC and *Bmal1-ELuc*, which were expected based on previous investigation^12^,^14^. The *ex vivo* circadian peak was slightly but significantly phase-advanced as compared with the *in vivo* peak by approximately 3 hours, regardless of the clock genes. The reason for these phase-advance shifts is unknown, but preparation of SCN slices for culturing could influence the circadian rhythm in culture^15^.

The *in vivo* monitoring technology enabled us to precisely analyze the temporal profiles of clock gene expression with respect to behavior outputs. Notably, this is the first demonstration of episodic bursts of clock gene reporters *in vivo*. Chi square periodogram revealed significant periodicities in the ultradian range, which was confirmed by wavelet analyses (Fig. 2, Supplementary Figure S2). Ultradian periodicity was also detected in activity bouts. However, the ultradian periods in two measures were slightly but significantly different, suggesting different origins.

Episodic bursts were often accompanied by activity bouts. An activity bout is known to be immediately followed by increases in the general circulation^16^ and in the brain temperature^17^,^18^. An increase in the circulation could enhance the entry of luciferin and other substances into the SCN neurons to affect the emission of bioluminescence. Episodic increases in the brain temperature could also affect the luciferin-luciferase enzymatic reaction. They could potentially serve as mechanisms of abrupt elevation of bioluminescence (artifact). However, the stoichiometric as well as the temporal analyses excluded the causality between the episodic burst of bioluminescence and activity bout (Figs 2,3 and 4). The circadian phase where episodic bursts occurred most robustly and frequently was not identical in PER2::LUC and *Bmal1-Eluc*; (Figs 1,3) with respect to phase-angle to the circadian behavior rhythm. The episodic bursts in *Bmal1-Eluc* was predominant in the sedative phase of animals (subjective day), whereas those in PER2::LUC was in the active phase of animals (subjective night). Discrepancy in the timings of the ultradian rhythm intensity was also shown by wavelet analysis in PER2::LUC and *Bmal1-ELuc* (Fig. 2, Supplementary Figure S2). In addition, causality is questioned by a lag time of 5 to 6 min reported between an activity bout and a following increase in the brain temperature^18^. Taking an almost simultaneous initiation of the episodic burst of bioluminescence and the activity bout into account, the increase in the brain temperature could not be a cause of an episodic burst of bioluminescence. Furthermore, there was no evidence for stoichiometric relationships between the episodic bursts and activity bouts (Fig. 4). Finally, some episodic bursts of bioluminescence lacked accompanying activity bouts (Fig. 2, Supplementary Figure S2). All these findings are against the idea of causality, and therefore the claim of artifact.

On the other hand, episodic bursts of bioluminescence and activity bouts frequently initiated simultaneously, suggesting a common trigger of both processes. Episodic bursts have also been demonstrated in multiple unit activity (MUA) of the SCN in freely moving animals^19^,^20^. Unlike our data, MUA episodes occurred when a behavior bout subsided. Causality between MUA and activity bout is not known. Although the origin of episodic bursts remains to be studied, the site of action is most likely intracellular processes. Recently, episodic bursts of gene transcription were demonstrated in cultured single cells across the mammalian genome^21^,^22^. The frequency and size of episodic bursts varied among genes and were modulated by transcriptional activators^22^,^23^. In the present study, the frequency and size of episodic bursts increased around the circadian peak phase of that gene, suggesting transcriptional activation is involved in the modulation of episodic bursts. However, episodic bursts have never been observed in cultured SCN slices (Fig. 1a), even when bioluminescence was measured at 1 min intervals (Supplementary Figure S4). The reason for this discrepancy is not known but could be explained by following mechanisms. Episodic bursts in dispersed single SCN cells, if any, could be cancelled by mutual couplings of single cells in the SCN tissue. Mutual couplings may synchronize individual circadian rhythms, which could obscure episodic bursts by smoothing. On the other hand, the discrepancy in episodic burst between the *in vivo* and *ex vivo* experiments could be explained by a shutdown of episodic inputs from outside the SCN. Episodic inputs of extra-SCN origin may trigger episodic changes of neuronal activity in the SCN cells, which ultimately synchronizes and/or enhances the episodic transcription of clock genes in the cells. Further studies are required to test this hypothesis.

In conclusion, clock gene expression in the SCN is under the regulation of two oscillatory mechanisms with different time scales, one is the circadian oscillation and the other is the ultradian rhythm with a period of approximately 3 h. The frequency and size of episodic bursts are modulated by the circadian rhythm of respective genes, indicating that clock gene expressions in the SCN are more dynamic *in vivo* than that expected from an auto-regulatory feedback loop for the circadian rhythm generation. There was no evidence for causality between episodic bursts of bioluminescence and often accompanying activity bouts.

## Methods

### Animals

Mice carrying a *Per1-luc*^11^ (hetero or homozygous), PER2::LUC^12^ (homozygous) or *Bmal1-ELuc*^24^ (heterozygous) reporter gene on a C57BL/6j background were used. PER2::LUC mice were originally produced in the Northwestern University. The animals had been backcrossed with C57BL/6j background mice (supplied from CREA Japan, Inc.) more than 10 generations when arrived at our laboratory. *Per1-luc* and *Bmal1-ELuc* mice were produced in YS New Technology Institute (Tochigi, Japan) and Institute of Advanced Industrial Science and Technology (Tsukuba, Japan), respectively. They were generated from fertilized eggs of C57BL/6j background; though created on a C57BL/6j background, we back-crossed these mice with C57BL/6j background mice as well for more than 7 generations. The mice were reared in our animal quarters where environmental conditions were controlled (light-dark cycle (LD); lights-on 6:00–18:00; light intensity approximately 100 lx at the cage bottom; humidity 60 ± 10%). Food and water were available ad libitum. Sterilized wooden chips were placed on the floor of an animal cage. Experiments were conducted in compliance with the rules and regulations established by the Animal Care and Use Committee of Hokkaido University under the ethical permission of the Animal Research Committee of Hokkaido University (Approval No. 08-0279).

### Behavioral activity measurement

Male and female mice were used in the present study at 2–6 months old (3.9 ± 1.4 months old, mean and SD). Mice were individually housed in polycarbonate cages (115 mm wide, 215 mm long, 300 mm high), and placed in a light-tight and air-conditioned box (40 × 50 × 50 cm). Spontaneous movements were measured by a passive infrared sensor which detects a change in thermal radiation from an animal due to behavior activity^25^. The amount of behavior activity was automatically recorded every min by computer (The Chronobiology Kit; Stanford Software System).

### Surgery

Surgical operation was performed under isoflurane anesthesia. A handmade guide cannula (inside diameter 1.12 mm, outer diameter 1.48 mm) was stereotaxically inserted into the brain (0.2 mm posterior and 0.2 mm lateral to the bregma, and 3.0 mm from the surface of the skull) and fixed by a dental resin. After a recovery period of more than 4 days, a polymethyl methacrylate optical fiber was inserted (fiber diameter, 0.5 mm; surface cladding, 0.25 mm thick) into the guide cannula aimed at the SCN (5.8 mm from the surface of the skull) and fixed with dental resin (Supplementary Figure S1b, c). More than 4 days later, an osmotic pump (flow speed, 0.5 μl/hr; pump volume, 200 μl; 2002, Alzet) containing D-luciferin K (100 mM) in saline was implanted in the peritoneal cavity for recording of *Per1-luc* and *Bmal1-ELuc*. For PER2::LUC recording, an osmotic pump (flow speed, 0.11 μl/hr; pump volume, 100 μl; 1004, Alzet) was implanted subcutaneously at the shoulders to deliver D-luciferin Na (50 mM) into the lateral ventricle. D-luciferin Na was dissolved in artificial cerebrospinal fluid (ACSF). A catheter connected to an osmotic pump was passed under the skin to the top of the head and inserted in to the lateral ventricle through a guide tube implanted beforehand at 0.6 mm posterior to the bregma, 1.4 mm to the lateral from the midline and 2.2 mm in depth.

Three to five days after the last surgery, bioluminescence recording began in constant darkness (DD). At the end of bioluminescence measurement, mice were transcardially perfused with physiological saline solution followed by 4% paraformaldehyde in 0.1 M phosphate buffer (PB) under ether anesthesia. Brains were cryoprotected in 20% sucrose in 0.1 M PB. Serial coronal sections (30 μm) were made by Cryostat (Leica) and stained with cresyl violet to identify the location of the optical fiber tip.

### *In vivo* bioluminescence measurement

*In vivo* measurement of bioluminescence was performed under DD to exclude the penetration of environmental lights. The day of release into DD was defined as Day 0. Bioluminescence emitted from the SCN was counted every minute via an optical fiber which was connected to a photon counting device (*In vivo* Kronos, Atto) equipped with a PMT (Hamamatsu Photonics). When bioluminescence was not detected in the first several days of measurement, the recording was stopped.

### SCN slice preparation

In the *ex vivo* experiment, mice reared in LD were euthanized by cervical dislocation and decapitated. The brain was rapidly removed and 300 μm coronal slices including the SCN was made by a microslicer (D.S.K: DTK-1000; Dosaka EM) in cooled Hanks’ Balanced salt solution (SIGMA). A trimmed slice of bilateral SCN was placed on a culture membrane (Milicell-CM, Millipore Corporations) in a 35-mm Petri dish. The slice was cultured in air at 36.5 °C with 1.2 ml Dulbecco’s modified Eagle’s medium (Invitrogen) with 0.1 mM D-luciferin K and 5% supplement solution, the composition of which was described previously^26^. Bioluminescence from the SCN slices was measured for 1 min at 10-min intervals or 1min intervals with a luminometer (Lumicycle, Actimetrics or Kronos, Atto). The day of slice preparation was defined as day 0.

To examine the spatial distribution of bioluminescence intensity in the SCN and peri-SCN regions, image records of the cultured SCN were obtained with a CCD camera and analyzed on a pixel level. Coronal SCN tissue slices were prepared from the mice carrying the same reporter genes used in the *in vivo* experiment. The analyses of SCN images were done at the circadian peak phase.

### Data analysis

Bioluminescence and behavior values were integrated in 5 min bins and the time series record of both measures were analyzed from Day 1 (1^st^ circadian cycle) to Day 5 (5^th^ circadian cycle) under DD. The onset of the 1^st^ circadian cycle was assigned to 24 h in local time (CT18) of Day 0. Significance of periodicity in the ultradian as well as circadian range was evaluated by Chi square periodogram (p < 0.05). Chi square periodogram for ultradian period was conducted after subtracting the circadian fluctuation from the original data. The circadian fluctuation was calculated by a 4 h moving average method. In most mice examined, significant peaks were detected at two to three ultradian ranges which seem more or less common to them. We calculated the mean value at the period ranges with the largest Qp value and regarded it as the major ultradian period. Wavelet analysis was applied to evaluate ultradian rhythms and periodic changes in the intensity of ultradian rhythms in a range from 0 to 12 hours^27^, using MatLab software. The bandwidth parameter was set to 3 and the center frequency to 1. Evaluation of rhythmicity and determination of periods were done by Chi square periodogram. Circadian periods of behavior rhythm were calculated by Chi square periodogram and from the slope of a regression line fitted to activity onsets.

Peak phases of circadian clock gene rhythms were calculated on Day 1 in DD and in culture by a geometric method described previously^28^. The activity onset was determined by ClockLab software (Actimetrics) for the calculation of circadian time (CT), where the activity onset was defined as CT12. When the results of *in vivo* and *ex vivo* experiments were compared, the local time was used as a common time reference.

Episodic bursts of bioluminescence were analyzed for their periodicity, dependency of occurrence (frequency) and of the size (amplitude and duration) on the circadian rhythm, and stoichiometric as well as temporal relation to activity bouts that often accompanied episodic bursts. An episodic burst of bioluminescence was defined as a bioluminescence increase whose amplitude was larger than 2 times of the mean level in the 5 day recording for at least 30 min. An episodic increase of behavior activity (activity bout) was defined as an abrupt increase in behavior activity from 0 counts, lasting at least 30 min. The magnitude of activity bout should be more than twice as large as the mean 30 min activity in the whole series of recording. The duration of burst or bout was defined as a length from the initial trough (bioluminescence) or the bin of 0 counts (behavior) to the end trough where the value lowered below the initial trough in a burst or became 0 counts in a bout. The peak latency of the episode was defined as the interval from the initial trough to the peak of the episodic burst or the activity bout. The size of the episodic burst was expressed by the area under the curve (AUC) which was calculated by summing bioluminescence above the baseline connecting the initial and end trough.

Stoichiometric as well as temporal analyses were carried out to examine potential causality between the episodic burst of bioluminescence and the activity bout. The mean number of occurrence as well as AUC of episodic bursts and activity bouts were assigned to 4 circadian phases (CT0-6, CT6-12, CT12-18, CT18-24) using the circadian peak as the phase reference. The analysis was performed for each clock gene reporter. Stoichiometric analysis was carried out for pairs of episodic bursts and activity bouts. In these analyses, only sporadic episodes or activity bouts which subsided within 60 min were used to exclude complexity due to overlapping episodes or bouts. Since an episodic burst was not necessarily accompanied by an activity bout, two different methods were introduced for analysis. First, bioluminescence data were averaged in reference to an activity bout (activity bout reference). Second, activity data were averaged in reference to an episodic burst (bioluminescence burst reference). Bioluminescence in individuals was calculated by subtracting the baseline values. The individual mean values were calculated first and then the mean of individual means were obtained for each reporter. The total number of activity bouts examined was 140 for *Per1-luc*, 51 for PER2::LUC and 113 for *Bmal1-ELuc*, and the total number of episodic bursts was 175 for *Per1-luc*, 35 for PER2::LUC and 145 for *Bmal1-ELuc*. Correlation analysis was performed with respect to AUC and the peak level for the pairs of activity bout and accompanying bioluminescence and for the pairs of episodic burst of bioluminescence and accompanying behavior activity.

### Statistics

Student’s t-test was used when two independent group means were compared. Welch’s t-test was used when the variances of two group means were different. A one-way repeated measure ANOVA with a post-hoc Tukey-Kramer was used to analyze a single time series data. A two-way factorial ANOVA with a post-hoc t-test was used when two independent time series data were compared (Statview or Statcel 3).

## Additional Information

**How to cite this article**: Ono, D. *et al.* Circadian and ultradian rhythms of clock gene expression in the suprachiasmatic nucleus of freely moving mice. *Sci. Rep.* **5**, 12310; doi: 10.1038/srep12310 (2015).

## Supplementary Material

Supplementary Information

## References

[b1] Reppert S.M. & Weaver D.R. Coordination of circadian timing in mammals. Nature 418, 935–941 (2002). 12198538 10.1038/nature00965

[b2] Darlington T.K *et al.* Closing the circadian loop: CLOCK-induced transcription of its own inhibitors per and tim. Science 280, 1599–1603 (1998). 9616122 10.1126/science.280.5369.1599

[b3] Shigeyoshi Y. *et al.* Light-induced resetting of a mammalian circadian clock is associated with rapid induction of the mPer1 transcript. Cell 91, 1043–1053 (1997). 9428526 10.1016/s0092-8674(00)80494-8

[b4] Yamanaka Y., Honma S. & Honma K. Daily exposure to a running wheel entrains circadian rhythms in mice in parallel with development of an increase in spontaneous movement prior to running-wheel access. Am. J. Physiol. Regul. Integr. Comp. Physiol. 305, R1367–1375 (2013). 24108869 10.1152/ajpregu.00389.2013

[b5] Yamaguchi S. *et al.* Synchronization of cellular clocks in the suprachiasmatic nucleus. Science 302, 1408–1412 (2003). 14631044 10.1126/science.1089287

[b6] Liu A.C. *et al.* Intercellular coupling confers robustness against mutations in the SCN circadian clock network. Cell 129, 605–616 (2007). 17482552 10.1016/j.cell.2007.02.047 PMC3749832

[b7] Yamaguchi S. *et al.* View of a mouse clock gene ticking. Nature 409, 684 (2001). 11217850 10.1038/35055628

[b8] Deisseroth K. Brain tissue light transmission calculator . (2015) Available at: http://web.stanford.edu/group/dlab/optogenetics/ (Accessed: 7^th^ March 2015).

[b9] Abe H. *et al.* Clock gene expressions in the suprachiasmatic nucleus and other areas of the brain during rhythm splitting in CS mice. Brain Res. Mol. Brain Res. 87, 92–99 (2001). 11223163 10.1016/s0169-328x(00)00295-3

[b10] Nishide S.Y. *et al.* New reporter system for Per1 and Bmal1 expressions revealed self-sustained circadian rhythms in peripheral tissues. Genes Cells 11, 1173–1182 (2006). 16999737 10.1111/j.1365-2443.2006.01015.x

[b11] Inagaki N., Honma S., Ono D., Tanahashi Y. & Honma K. Separate oscillating cell groups in mouse suprachiasmatic nucleus couple photoperiodically to the onset and end of daily activity. Proc. Natl. Acad. Sci. USA 104, 7664–7669 (2007). 17463091 10.1073/pnas.0607713104 PMC1857228

[b12] Yoo S.H. *et al.* PERIOD2::LUCIFERASE real-time reporting of circadian dynamics reveals persistent circadian oscillations in mouse peripheral tissues. Proc. Natl. Acad. Sci. USA 101, 5339–5346 (2004). 14963227 10.1073/pnas.0308709101 PMC397382

[b13] Noguchi T. *et al.* Dual-color luciferase mouse directly demonstrates coupled expression of two clock genes. Biochemistry 49, 8053–8061 (2010). 20718447 10.1021/bi100545h

[b14] Myung J. *et al.* Period coding of Bmal1 oscillators in the suprachiasmatic nucleus. J. Neurosci. 32, 8900–8918 (2012). 22745491 10.1523/JNEUROSCI.5586-11.2012 PMC6622328

[b15] Yoshikawa T., Yamazaki S. & Menaker M. Effects of preparation time on phase of cultured tissues reveal complexity of circadian organization. J. Biol. Rhythms 20, 500–512 (2005). 16275769 10.1177/0748730405280775 PMC1470468

[b16] Sheward W. J. *et al.* Circadian control of mouse heart rate and blood pressure by the suprachiasmatic nuclei: behavioral effects are more significant than direct outputs. PLoS ONE 5, e9783. 20339544 10.1371/journal.pone.0009783 PMC2842429

[b17] Baker F.C., Angara C., Szymusiak R. & McGinty D. Persistence of sleep-temperature coupling after suprachiasmatic nuclei lesions in rats. Am. J. Physiol. Regul. Integr. Comp. Physiol. 289, R827–838 (2005). 15860650 10.1152/ajpregu.00093.2005

[b18] Ootsuka Y. *et al.* Brown adipose tissue thermogenesis heats brain and body as part of brain-coordinated ultradian basic rest-activity cycle. Neuroscience 164, 849–861 (2009). 19679172 10.1016/j.neuroscience.2009.08.013 PMC2767384

[b19] Meijer J.H., Schaap J., Watanabe K. & Albus H. Multiunit activity recordings in the suprachiasmatic nuclei: *in vivo* versus *in vitro* models. Brain Res. 753, 322–327 (1997). 9125419 10.1016/s0006-8993(97)00150-9

[b20] Nakamura W. *et al.* *In vivo* monitoring of circadian timing in freely moving mice. Curr. Biol. 18, 381–385 (2008). 18334203 10.1016/j.cub.2008.02.024

[b21] Suter M.D. *et al.* Mammalian genes are transcribed with widely different bursting kinetics. Science 332, 472–474 (2011). 21415320 10.1126/science.1198817

[b22] Dar D.R. *et al.* Transcriptional burst frequency and burst size are equally modulated across the human genome. Proc. Natl. Acad. Sci. USA 109, 17454–17459 (2012). 23064634 10.1073/pnas.1213530109 PMC3491463

[b23] Senecal A. *et al.* Transcriptional factors modulate c-Fos transcriptional bursts. Cell Rep. 8, 75–83 (2014). 24981864 10.1016/j.celrep.2014.05.053 PMC5555219

[b24] Nakajima Y. *et al.* Enhanced beetle luciferase for high-resolution bioluminescence imaging. PLoS ONE 5, e10011 (2010). 20368807 10.1371/journal.pone.0010011 PMC2848861

[b25] Abe H., Honma S., Ohtsu H. & Honma K. Circadian rhythms in behavior and clock gene expressions in the brain of mice lacking histidine decarboxylase. Brain Res. Mol. Brain Res. 124, 178–87 (2004). 15135226 10.1016/j.molbrainres.2004.02.015

[b26] Ono D., Honma S. & Honma K. Cryptochromes are critical for the development of coherent circadian rhythms in the mouse suprachiasmatic nucleus. Nat. Communs. 4, 1666 10.1038/ncomms2670. (2013). 23575670

[b27] Araszkiewicz A. & Bogusz J. Application of wavelet technique to the Earth tides observations analyses. Marees Terrestres Bulletin d’Informations (BIM) . 146, 11789–11798 (2010).

[b28] Hashimoto S., Nakamura K., Honma S., Tokura H. & Honma K. Melatonin rhythm is not shifted by lights that suppress nocturnal melatonin in humans under entrainment. Am. J. Physiol. 270, R1073–1077 (1996). 8928908 10.1152/ajpregu.1996.270.5.R1073

