# Supplementary material for: Circadian and ultradian rhythms of clock gene expression in the suprachiasmatic nucleus of freely moving mice
Source: Sci Rep. 2015 Jul 21;5:12310. doi: 10.1038/srep12310 (PMC4508664; doi:10.1038/srep12310)
Supplement: Supplementary Information [file srep12310-s1.pdf]

Circadian and ultradian rhythms of clock gene expression in the suprachiasmatic nucleus of freely moving mice

Daisuke Ono[1], Ken-ichi Honma*[2], and Sato Honma*[2]

[1]Photonic Bioimaging Section, Research Center for Cooperative Projects, Hokkaido University Graduate School of Medicine, Sapporo, 060-8638, Japan
[2]Department of Chronomedicine, Hokkaido University Graduate School of Medicine, Sapporo, 060-8638, Japan

*Correspondence to: kenhonma@med.hokudai.ac.jp and sathonma@med.hokudai.ac.jp

Supplementary information

Supplementary figure legends

Supplementary Figure S1 *In vivo* monitoring of clock gene expressions in the SCN

(a) Set-up for *in vivo* recording: a photon counting device (left) and a light-tight box for *in vivo* monitoring (right). (b) A mouse with an implanted optical fiber. (c) Photomicrograph of the SCN stained with cresyl violet after recording of bioluminescence. The tip of the optical fiber was located just above of the SCN. Scale bar is 1.0 mm. (d) Background noise levels in the conventional PMT (black) without cooling and the optimized PMT (red) cooled to 10 ºC. The noise levels were measured

without connecting an optical fiber. (e) A typical example of bioluminescence (blue) and behavior (black) records for 3 weeks in a freely moving mouse carrying a *Per1-luc* reporter. Circadian rhythms in both parameters are illustrated by a double plotted actogram. Bioluminescence and activity in 5 min bins are plotted as vertical bars across each day, and consecutive days are plotted from top to bottom. Bioluminescence data are smoothed by a 4 h moving average method and detrended by a 24 h moving average subtraction method. A yellow star indicates the time of a light pulse exposure and a red arrow indicates the time of osmotic pump exchange under isoflurane anesthesia.

Supplementary Figure S2 Circadian and episodic fluctuations of clock gene expression

(a, d, g) Other examples of *in vivo* circadian gene expression rhythms superimposed by episodic bursts are illustrated with colored lines for 2 cycles in DD (a, *Per1-luc*; d, PER2::LUC; g, *Bmal1-ELuc*). Raw data (non-detrended and non-smoothed) are plotted in 5 min bins. Behavior activity in 5 min bins is indicated by black lines. Though animals were housed in DD, 06:00 and 18:00 local time are shown for reference by the vertical solid and broken lines, respectively. (b, e, h) Wavelet spectrum in the ultradian range (0-12 h) are illustrated for episodic bursts of bioluminescence (upper) and for

activity bout (lower) for each clock gene. The intensity of rhythm at a particular period was expressed with different colors. Dark red indicates the intensity larger than 1.5 time of the individual mean peak level of bioluminescence burst. Intensities below this level are expressed by different colors down to dark blue. (c, f, i) Chi square periodograms in the circadian (upper) and ultradian range (lower) are demonstrated for bioluminescence (left) and behavior activity (right) in each clock gene. An oblique line in each periodogram indicates a significance level of p=0.05.

Supplementary Figure S3 Bioluminescence emitted from the cultured SCN

Spatial distribution of bioluminescence intensity emitted from the SCN and peri-SCN regions *ex vivo* are illustrated for each clock gene reporter. Changes in the intensity along the dorsoventral and the mediolateral axis (shown by yellow lines) were expressed as an intensity map across all pixels on the lines (top and right plots). The yellow lines intersect the brightest portion in the SCN slice. The bioluminescence intensity decreased rapidly with distance from the SCN border indicated by a white broken line in each picture. The light intensity at the border on a yellow line is indicated by a crossing point of a broken and a distribution line in each intensity map. The intensity at the peri-SCN of dorsal border was less than 10 % of the brightest part in the

SCN.

Supplementary Figure S4 Circadian rhythms of the cultured SCN plotted at 1min intervals

Circadian bioluminescence rhythms from cultured SCN slice from *Per1-luc* (a), PER2::LUC (b), and *Bmal1-ELuc* (c) mice. Raw data (non-detrended, non-smoothed) were illustrated at 1 min intervals for 2 successive days.

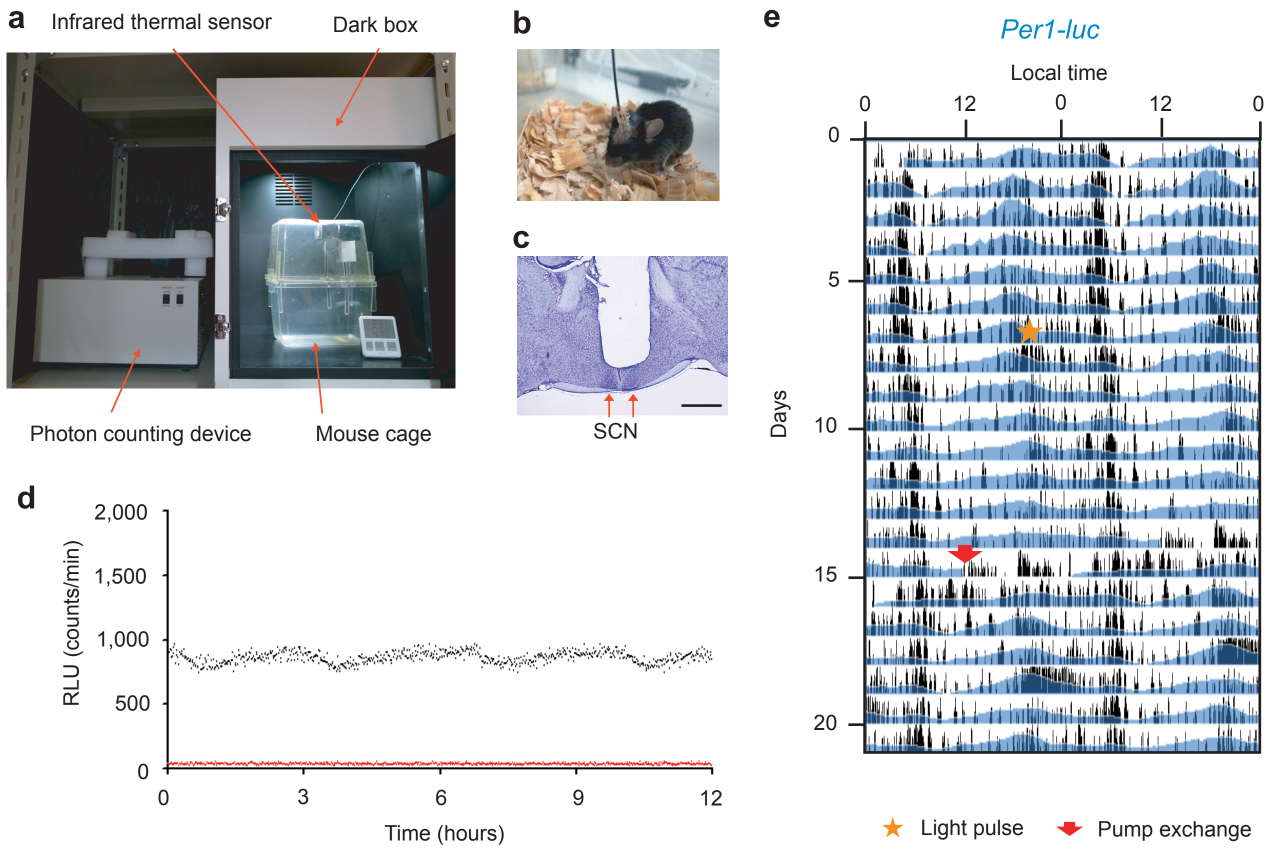

Supplementary figure 1

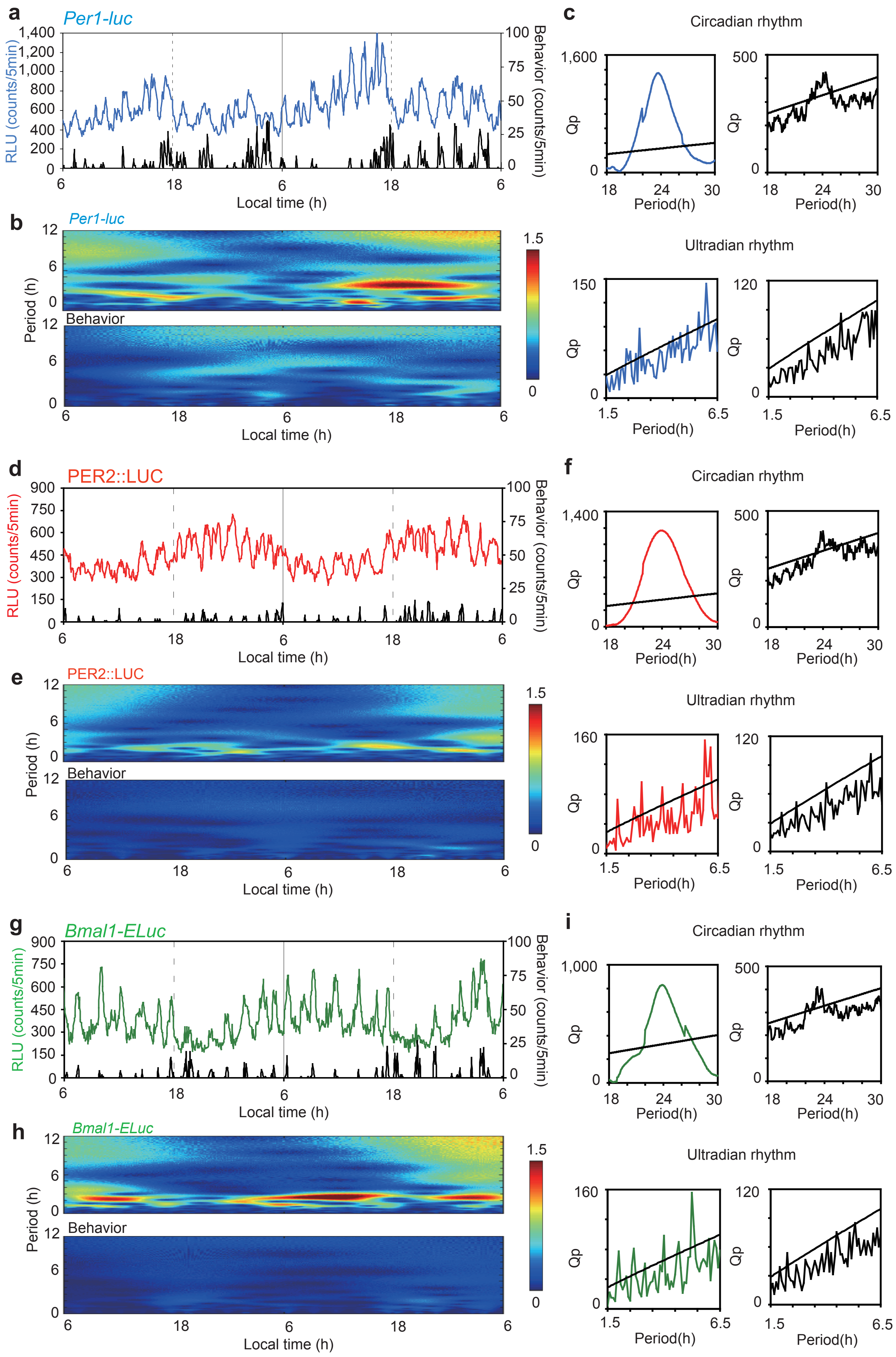

Supplementary figure 2

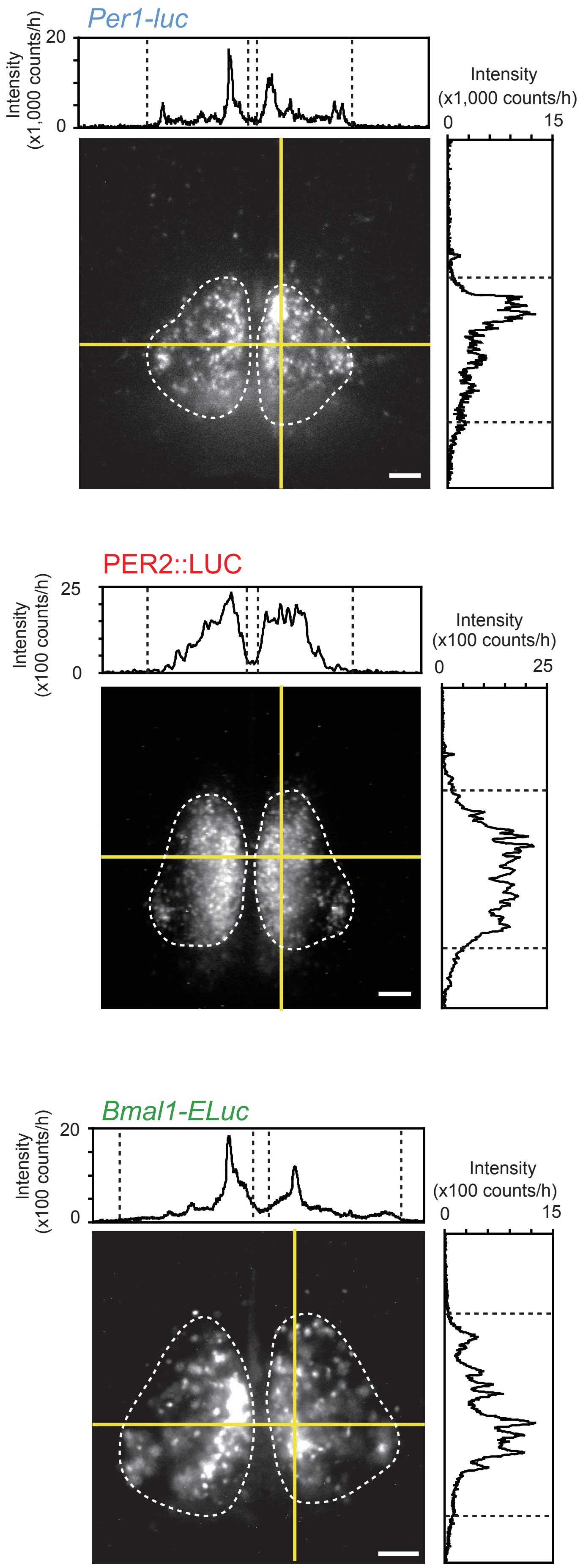

Supplementary figure 3

Ex vivo

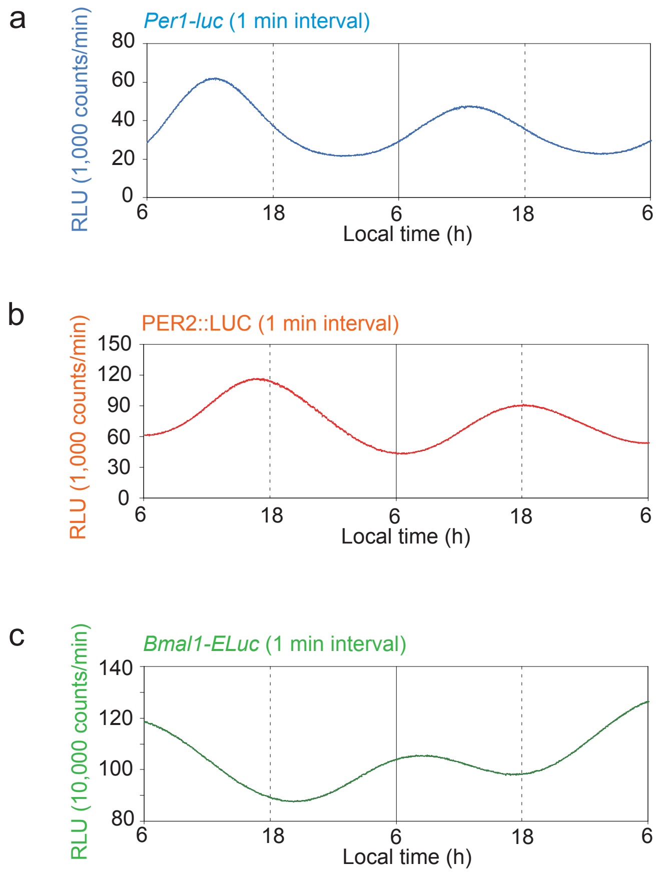

Supplementary figure 4
